# Supplementary material for: Development of a Microfluidic Device to Form a Long Chemical Gradient in a Tissue from Both Ends with an Analysis of Its Appearance and Content
Source: Micromachines (Basel). 2021 Nov 29;12(12):1482. doi: 10.3390/mi12121482 (PMC8709218; doi:10.3390/mi12121482)
Supplement: Supplementary file 1 [file micromachines-12-01482-s001.zip › micromachines-1464195-supplementary.pdf]

Supplementary Materials of "Development of Microfluidic Device to Form Long Chemical Gradient in a Tissue from Both Ends with Analysis of its Appearance and Content"

Yasunori Tokuoka*, Keiichi Kondo, Noboru Nakaigawa and Tadashi Ishida*

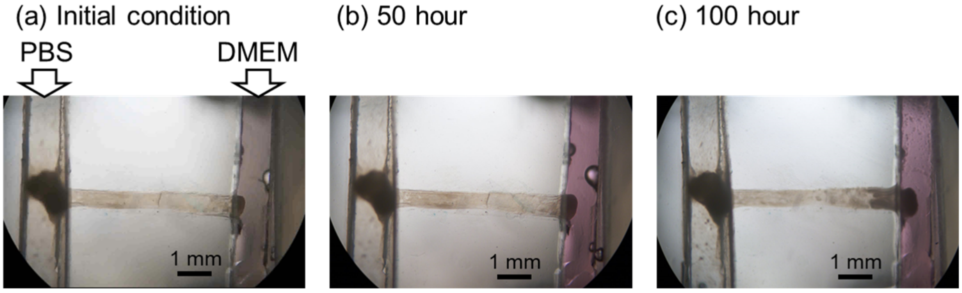

Figure S1. One-hundred-hour perfusion experiment using phosphate-buffered saline (PBS) and Dulbecco's modified Eagle medium (DMEM). (a) initial condition, (b) 50 hours and (c) 100 hours later.

Table S1. Leakage test through the connection channel with different size of simulated tissues. The cross section of the connection channel was circle of 0.5 mm in diameter.

Diameter of silicone tissue [mm]	0.29	0.31	0.35	0.37	0.46
Diameter of pillar / diameter of connection channel	0.58	0.62	0.71	0.74	0.92
Leakage	N.D.	N.D.	N.D.	N.D.	N.D.

N.D. not detected
